# Neutral lipid storage disease with myopathy in China: a large multicentric cohort study

**DOI:** 10.1186/s13023-019-1209-z

**Published:** 2019-10-26

**Authors:** Wei Zhang, Bing Wen, Jun Lu, Yawen Zhao, Daojun Hong, Zhe Zhao, Cheng Zhang, Yuebei Luo, Xueliang Qi, Yingshuang Zhang, Xueqin Song, Yuying Zhao, Chongbo Zhao, Jing Hu, Huan Yang, Zhaoxia Wang, Chuanzhu Yan, Yun Yuan

**Affiliations:** 1 Department of Neurology, Peking University First Hospital, No.8 Xishiku Street, West District, Beijing, 100034 China; 2 Department of Neurology, Qilu Hospital of Shandong University, No. 107, West Wenhua Road, Jinan, 250012 Shandong China; 3 Department of Neurology, Huashan Hospital of Fudan University, Shanghai, People’s Republic of China; 4 Department of Neurology, Peking University People’s Hospital, Beijing, People’s Republic of China; 5 Department of Neurology, the Third Hospital of Hebei Medical University, Shijiazhuang, Hebei province, People’s Republic of China; 6 Department of Neurology, the First Affiliated Hospital of Sun yat-sen University, Guangzhou, Guangdong province, People’s Republic of China; 7 Department of Neurology, Xiangya Hospital, Central South University, Changsha, Hunan province, People’s Republic of China; 8 Department of Neurology, the Second Affiliated Hospital of Nanchang University, Nanchang, Jiangxi province, People’s Republic of China; 9 Department of Neurology, Third Hospital, Peking University, Beijing, People’s Republic of China; 10 Department of Neurology, the Second Hospital of Hebei Medical University, Shijiazhuang, Hebei province, People’s Republic of China

**Keywords:** Neutral lipid storage disease with myopathy, Patatin-like phospholipase domain-containing 2, Rimmed vacuole, Skeletal myopathy, Cardiomyopathy

## Abstract

**Background:**

Neutral lipid storage disease with myopathy (NLSDM) is a rare clinical heterogeneous disorder caused by mutations in the patatin-like phospholipase domain-containing 2 (PNPLA2) gene. NLSDM usually presents skeletal myopathy, cardiomyopathy and the multiple organs dysfunction. Around 50 cases of NLSDM have been described worldwide, whereas the comprehensive understanding of this disease are still limited. We therefore recruit NLSDM patients from 10 centers across China, summarize the clinical, muscle imaging, pathological and genetic features, and analyze the genotype-phenotype relationship.

**Results:**

A total of 45 NLSDM patients (18 men and 27 women) were recruited from 40 unrelated families. Thirteen patients were born from consanguineous parents. The phenotypes were classified as asymptomatic hyperCKemia (2/45), pure skeletal myopathy (18/45), pure cardiomyopathy (4/45), and the combination of skeletal myopathy and cardiomyopathy (21/45). Right upper limb weakness was the early and prominent feature in 61.5% of patients. On muscle MRI, the long head of the biceps femoris, semimembranosus and adductor magnus on thighs, the soleus and medial head of the gastrocnemius on lower legs showed the most severe fatty infiltration. Thirty-three families were carrying homozygous mutations, while seven families were carrying compound heterozygous mutations. A total of 23 mutations were identified including 11 (47.8%) point mutations, eight (34.8%) deletions and four (17.4%) insertions. c.757 + 1G > T, c.245G > A and c.187 + 1G > A were the three most frequent mutations. Among four groups of phenotypes, significant differences were shown in disease onset (< 20 years versus ≥20 years old, *p* = 0.003) and muscle pathology (with rimmed vacuoles versus without rimmed vacuoles, *p* = 0.001). PNPLA2 mutational type or functional defects did not show great impact on phenotypes.

**Conclusion:**

We outline the clinical and genetic spectrum in a large cohort of NLSDM patients. Selective muscle fatty infiltration on posterior compartment of legs are characteristic of NLSDM. Chinese patients present with distinctive and relative hotspot PNPLA2 mutations. The disease onset age and pathological appearance of rimmed vacuoles are proved to be related with the clinical manifestations. The phenotypes are not strongly influenced by genetic defects, suggesting the multiple environmental risk factors in the development of NLSDM.

## Background

Neutral lipid storage disease (NLSD) includes two autosomal recessive disorders. Neutral Lipid Storage Disease with Myopathy (NLSDM; MIM 610717) is caused by patatin-like phospholipase domain-containing 2 (PNPLA2/ATGL) mutations [1], while Neutral Lipid Storage Disease with Ichthyosis (MIM 604780) or Chanarin-Dorfman syndrome is caused by comparative gene identification-58 (CGI-58/ABHD5) mutations [2]. NLSDM usually affects the skeletal and cardiac muscles, with occasional involvement of the liver, pancreas or brain [3–5]. NLSDM mainly occurs in the early 30s [1, 3–5], and is rare before 20 years of age [4, 6, 7]. Clinical phenotypes vary from hyperCKemia [6, 8], limb girdle/distal myopathy with [1, 9–11] or without cardiomyopathy [10, 12, 13] to the rarely reported pure cardiomyopathy [14]. Muscle magnetic resonance imaging (MRI) shows asymmetric fatty infiltration predominantly involving the posterior compartment of the limb muscles [8, 11–17]. The pathology commonly shows lipid droplets with or without rimmed vacuoles (RVs) in myofibers, and Jordan’s anomaly in peripheral blood smear [1, 4, 5, 12]. PNPLA2 mutations are mainly located on the C-terminal, and tend to cluster in exons 4 to 7 [3–5, 18]. A wide range of intra-familial phenotypic variability also appears in subjects carrying the same mutations [4, 10, 19].

Approximately 50 cases of NLSDM have been reported worldwide from a range of various ethnic groups [1, 3–5, 8, 14, 20] including Chinese [10, 12, 21, 22]. However, NLSDM patients are typically described from a few families in case reviews [1, 3, 4, 8, 14, 20] or in small cohorts of patients [4, 5, 16]. The rarity and broad geographical distribution of NLSDM limits a comprehensive understanding and prompt diagnosis of this disease, and the phenotype/genotype correlation remains unclear [3, 4, 15, 20].

In the present study, we recruited NLSDM patients from neuromuscular centers across China. We examined the clinical profiles, fatty replacement pattern using lower leg muscle MRI, PNPLA2 mutational features and the phenotype/genotype correlation in this series.

## Methods

### Patient registry

Ten centers across China agreed to take part in the NLSDM registry. The diagnosis was pathogenic PNPLA2 gene variations, with Jordan’s anomaly in blood smears or lipid storage within muscle fibers. Clinical data collection included age of disease onset, gender, nationality, the duration from onset to diagnostic time, initial symptoms, skeletal muscle weakness distribution, heart and other multiple organs dysfunction, creatine kinase level, electrophysiological findings, electrocardiogram findings, ultrasonography and MRI of the heart. All patients were classified clinically according to their onset age (< 20 or ≥ 20 years old) and their phenotypes (involvement of skeletal muscle or cardiac muscles).

### Muscle MRI

Muscle MRI at 3.0 T (GE 1.5 Sigma Twin Speed; GE Healthcare, Waukesha, WI, USA) was performed at both axial and coronal sections on the thighs and calves. The gluteus maximus was observed at the pelvic level. The rectus femoris, vastus lateralis, vastus medialis, vastus intermedius, sartorius, gracilis, adductor longus, adductor magnus, long head of the biceps femoris, semitendinosus and semimembranosus were observed at the mid-thighs. The anterior tibialis, posterior tibialis, extensor digitorum longus, peroneus longus and brevis, soleus and medial and lateral head of gastrocnemius were observed at the mid-calves. Axial T1-weighted spin echo series with 450/12 (repetition time, ms/echo time, ms) was used for semi quantitative evaluation of fatty infiltration according to the modified Mercuri scale (0–5 scale) [23, 24]. Axial short TI inversion recovery series with 5000/90 (repetition time, ms/echo time, ms) were used to assess the degree of edema (0–5 scale) [23, 24]. For asymmetric muscles, we chose the score from the most severe side of the leg.

### Pathology

Open muscle biopsies were performed in some patients after consent forms were obtained. Tissue specimens were immediately frozen in cooled isopentane and stored in liquid nitrogen. Standard histological and histochemical techniques were applied to 10 μm frozen sections, including hematoxylin & eosin, modified Gomori trichrome, periodic acid Schiff reaction, oil red O, reduced nicotinamide adenine dinucleotide tetrazolium reductase, succinate dehydrogenase, cytochrome-c oxidase reaction, adenosine triphosphatase (at pH 4.3, 4.6 and 9.4) and nonspecific esterase. In some patients, part of the specimen was fixed in 2% in glutaraldehyde at rest length and post-fixed in osmic acid for electron microscopy.

Peripheral blood smears were stained with Wright‘s or oil red O to observe Jordan’s anomaly by light microscopy.

### Gene testing

Genomic DNA from peripheral blood was extracted for direct sanger sequencing of the coding exons and exon/intron boundaries on PNPLA2 gene (in 8 famiies including F4, F9–13, F20, F22) or targeted next-generation sequencing covering 650 genes related to hereditary neuromuscular disorders (in the remaining 32 families). The coverage of PNPLA2 gene in these patients was 100% on the depth of 10X, 99.8 to 100% on the depth of 20X, and 95.8 to 100% on the depth of 50X. If the variants were identified by next-generation sequencing, sanger sequencing was used to confirm the findings. The blood samples were collected from the parents to clarify the variant origin and test co-separation. The genetic variants were assessed by the Exome Aggregation Consortium, Genome Aggregation Database and 1000 Genomes project (1000G). The effect of missense variants was predicted by PolyPhen2, Mutation taster and SIFT software for in silico functional analysis. The variants were finally interpreted according to the American College of Medical Genetics and Genomics guidelines. The types, frequencies and locations of allelic variants were analyzed.

### Statistical analysis

Clinical phenotypes were compared between the multiple variables including gender (male versus female), disease age onset (< 20 years versus ≥20 years old), muscle pathology (with RVs versus without RVs), mutation type (homozygous versus heterozygous mutation) and mutation severity (with one or two null mutations versus missense/in-frame mutations). For statistical analysis, continuous variables were described as mean ± standard deviation for a normal distribution, or as median and interquartile range (IQR) range for a skewed distribution. Differences between the groups were tested using the chi square test. Statistically significance was considered as *p* < 0.05. All data were analyzed using statistical software SPSS 22.0 (SPSS, Inc., Chicago, IL, USA).

## Results

### Clinical characteristics

**Fig. 1:**
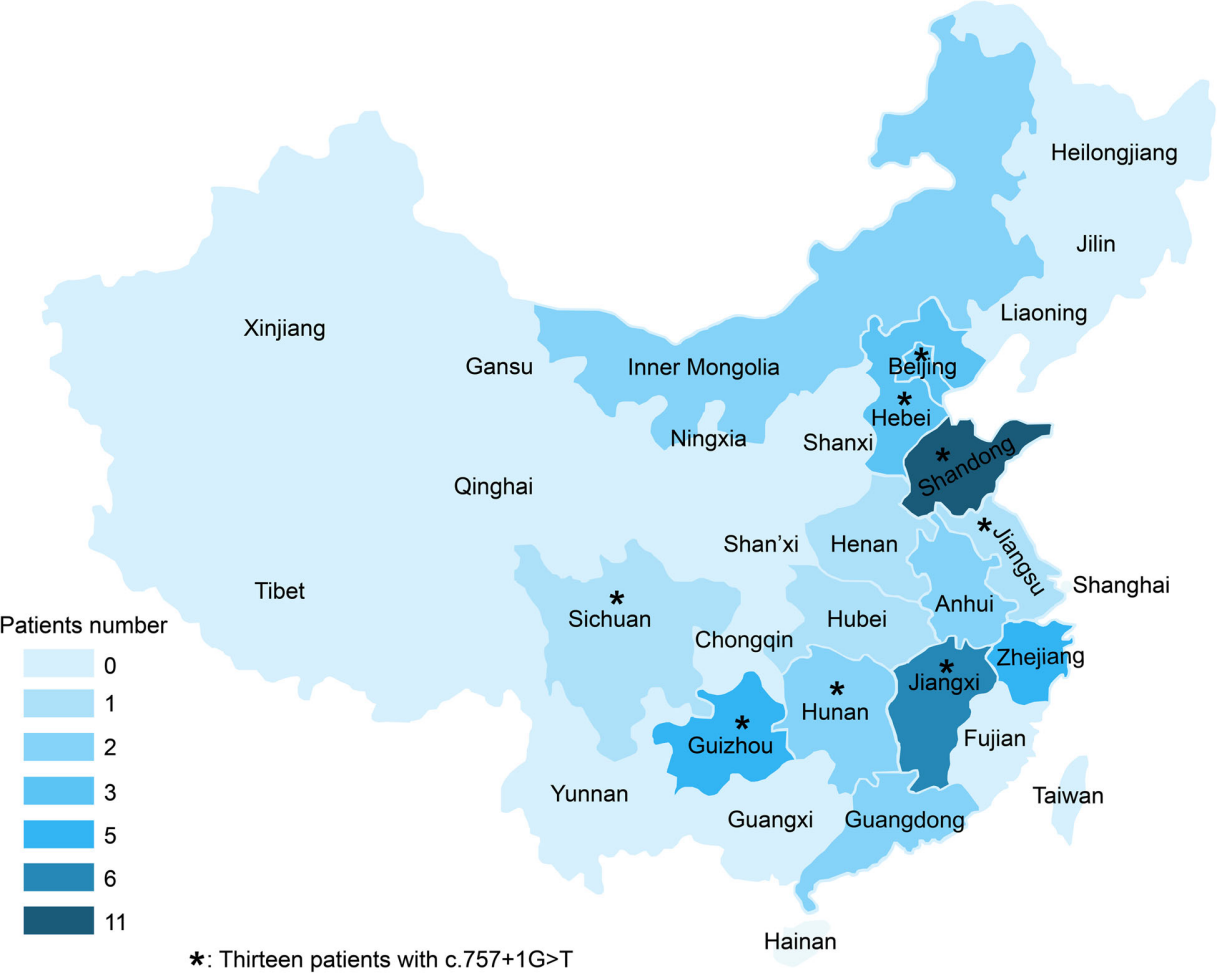
Geographical origin of 45 Chinese patients with NLSDM. The common mutation of *c.*757 + 1 G > T is marked with star

A total of 45 NLSDM patients (18 men and 27 women) were recruited from 40 unrelated families. All patients were from the Chinese mainland and distributed in fifteen provinces and municipalities, prevalent in eastern China (Fig. 1). Forty one patients were Han, two were Dong and two were Mongolian nationalities. Thirteen patients were born from consanguineous parents. The median (IQR) age of onset was 33 (26, 40) years old. Five patients had symptoms before 20 years of age, and 40 patients after 20 years of age. The median (IQR) time from onset to diagnosis was 6 (3, 9) years. The initial symptoms included limb weakness (36/45, 80.0%), exercise intolerance (2/45, 4.4%), hyperCKemia (2/45, 4.4%), palpitation (3/45, 6.7%), chest pain (1/45, 2.2%) and hearing loss (1/45, 2.2%). Of the 36 patients with limb weakness as first symptoms, 21 (58.3%) patients developed asymmetric weakness from the right arm (15 patients), right leg (3 patients) and right limbs (3 patients). The remaining 15 (41.7%) patients developed symmetric weakness from both arms (4 patients), legs (5 patients) and four limbs (6 patients). To the time of diagnosis, the clinical phenotypes were classified as pure skeletal myopathy (18/45, 40.0%), skeletal myopathy with cardiomyopathy (21/45, 46.7%), pure cardiomyopathy (4/45, 8.9%) and asymptomatic hyperCKemia (2/45, 4.4%).

In 39 patients with skeletal myopathy with or without cardiomyopathy, limb weakness appeared in all patients (100%). Neck flexion weakness appeared in 25/39 (64.1%) patients. No bulbar palsy or respiratory insufficiency was revealed. Muscle weakness predominantly involved the proximal limbs in 33/39 (84.6%) patients and the distal limbs in 6/39 (15.4%) patients. Asymmetric weakness was revealed in 24/39 (61.5%) patients, all of whom showed the most prominent involvement of the right arm. Symmetric limb weakness was noted in 15/39 (38.5%) patients, in whom nine showed the most prominent involvement of the arms and six of the legs. Diffuse muscle atrophy was noted in 30/39 (76.9%) patients and scapular winging in 22/39 (56.4%) patients. Other organ dysfunction included hearing loss (10, 22.2%), short stature (7, 15.6%), hypertension (6, 13.3%), diabetes mellitus (2, 4.4%), hepatomegaly (2, 4.4%), acute necrotizing pancreatitis (2, 4.4%), intellectual disability (1, 2.2%), cataract (1, 2.2%) and diarrhea (1, 2.2%).

Serum creatine kinase levels in 45 patients ranged from normal to 25 times of the upper limit (normal range: 25–170 IU/L). Needle electromyography revealed myopathic changes in 39/43 (90.7%) patients, neurogenic in one (2.3%) and normal in three (7.0%). Three (7.0%) cases showed myotonia discharge. Nerve conduction were found abnormal in 11/43 (25.6%) of the patients involving upper limbs in 6 (14.0%), lower limbs in 2 (4.7%) and both in 3 (7.0%) cases. Three (7.0%) cases showed multiple neuropathies. Compound muscle action potentials decreased in 8 cases and sensory nerve action potential decreased in one case. Conduction velocities were revealed decreasing in sensory nerves of two cases. H reflexes response of tibial nerves disappeared in three cases. Electrocardiography revealed abnormalities in 12/45 (26.7%) patients, including elevated ST-T segment in five, paroxysmal atrial fibrillation with left anterior fascicular block in one, nodal tachycardia in two, sinus bradycardia in two, non-sustained ventricular tachycardia in one and old myocardial infarction in one (p39) who did not reveal any artery stenosis on coronary angiography. Ultrasound cardiography revealed cardiomyopathy in 13/35 (28.9%) patients, including hypertrophic in ten and dilated cardiomyopathy in three, five of whom were further confirmed by cardiac MRI.

Details of clinical information was listed in Table 1.

**Table 1:** Clinical profiles, muscle pathology and PNPLA2 mutations from 45 neutral lipid storage disease with myopathy (NLSDM) patients

No. (Family)	S/A/D	Initial symptoms	Weakness Distribution	Severe limbs^a^	Subtype	JA	Muscle pathology	PNPLA2 mutations	Parental derivation	Domain
P1 (F1)	M/4/1	HyperCK	NA	NA	AH	+	Lipid	c.659_667del, *p.*Y220_L222del	P/M	
P2(F2)	F/4/22	EI	P	B/LL	S/C	+	Lipid	c.353 T > C *p.*L118P	M	PA
								c.356C > A, *p.*T119 N	P	PA
p3(F3)	F/46/3	B/LL	P	R/UL	S	+	Lipid/RVs	c.478_479 insCTCC, *p.*Q160Pfs^a^19	NA/NA	
P4(F4)	F/48/4	B/UL, LL	P	B/LL	S/C	+	Lipid/RVs	c.245G > A, p.G82D	M	PA
								*c.*564G > A, *p.*S188=	NA	
P5(F5)	F/29/6	Palpitation	NA	NA	C	+	Lipid	c.333_334del, *p.*S111Rfs^a^66	NA/NA	
P6(F6)	M/23/19	Palpitation	NA	NA	C	NA	Lipid	c.918del, *p.*A307Pfs^a^13	P/M	
P7(F7)	M/21/1	Chest pain	NA	NA	C	+	Lipid	c.757 + 1G > T	P/NA	
P8 (F8)	F/41/5	B/UL	P	B/UL	S	+	Lipid/RVs	c.245G > A, *p.*G82D	P/M	PA
P9 (F8)	M/33/5	Palpitation	NA	NA	C	+	Lipid	c.245G > A, *p.*G82D	P/M	PA
P10 (F9)	F/35/5	B/UL, LL	P	B/UL	S	+	Lipid/RVs	c.187 + 1G > A	P/M	
P11 (F9)	M/35/20	Hearing loss	D	B/LL	S/C	+	Lipid/RVs	c.187 + 1G > A	P/M	
P12(F10)	M/31/13	R/LL	P	R/UL	S/C	NA	Lipid	c.187 + 1G > A	P/M	
P13(F10)	M/27/15	R/UL	P	R/UL	S/C	+	Lipid/RVs	c.187 + 1G > A	P/M	
P14(F11)	M/41/3	R/UL, LL	P	R/UL	S/C	+	Lipid/RVs	c.757 + 2 T > C	NA	
								c.749A > C, *p.*Q250P	NA	
P15(F12)	F/45/3	R/UL	D	R/UL	S	+	Lipid/RVs	c.757 + 1G > T	NA/NA	
P16(F13)	M/38/1	R/UL	P	R/UL	S	+	Lipid	c.467del, *p.*P156Lfs^a^100	NA/NA	
P17(F14)	M/22/6	R/LL	D	B/LL	S	+	Lipid/RVs	c.434G > A, *p.*S145 N	NA/NA	PA
P18(F15)	F/42/7	R/UL	P	R/UL	S/C	+	Lipid/RVs	c.6_7insT, *p.*P3Sfs^a^62	NA/NA	
P19(F16)	M/34/1	R/UL, LL	P	B/UL	S/C	+	Lipid	c.475_476 insCTCC, L159Pfs^a^20	NA/NA	
P20(F17)	M/35/6	R/UL, LL	P	R/UL	S	+	Lipid/RVs	c.467del, *p.*P156Lfs^a^100	NA/NA	
P21(F18)	M/36/6	B/UL, LL	P	R/UL	S	+	Lipid/RVs	c.697-1G > A	NA/NA	
P22(F19)	M/3.5/0.17	HyperCK	NA	NA	AH	+	Lipid	c.516C > G *p.*N172K	M	PA
								c.918del, p.A307Pfs^a^13	P	
P23(F20)	F/34/7	R/UL, LL	P	R/UL	S/C	+	NA	c.291_292insT, *p.*L98Pfs^a^8	NA/NA	
P24(F21)	F/26/2	B/UL, LL	P	B/UL	S/C	NA	Lipid	c.757 + 1G > T	NA/NA	
P25(F22)	F/17/17	B/UL	P	B/UL	S/C	NA	Lipid	c.798delC, *p.*A267P fs^a^53	NA/NA	
P26(F23)	M/22/5	B/LL	P	R/UL	S/C	NA	Lipid	c.475_476 insCTCC, *p.*L159Pfs^a^20	NA/NA	
P27(F24)	F/30/11	B/LL	P	R/UL	S/C	NA	Lipid	c.757 + 1G > T	NA/NA	
P28(F25)	F/36/6	B/LL	P	R/UL	S/C	NA	Lipid	c.245G > A, *p.*G82D	NA/NA	PA
P29(F26)	F/40/10	B/UL	P	B/UL	S/C	+	Lipid	c.245G > A *p.*G82D	NA/NA	PA
								c.516 del *p.*L173C fs^a^83	NA/NA	
P30(F27)	F/40/13	R/UL	P	R/UL	S/C	+	Lipid/RVs	c.245G > A, *p.*G82D	NA/NA	PA
P31(F28)	F/32/9	B/UL	P	R/UL	S	+	Lipid/RVs	c.187 + 1G > A	P/M	
P32(F29)	F/29/4	R/UL	P	R/UL	S	+	Lipid/RVs	c.757 + 1G > T	P/M	
P33(F30)	M/37/3	R/UL	P	R/UL	S	NA	Lipid/RVs	c.757 + 2 T > C	P	
								c.749A > C, *p.*Q250P	M	
P34(F31)	F/42/9	R/UL	P	R/UL	S	+	Lipid/RVs	c.245G > A, *p.*G82D	P	PA/
								c.922 del, p.L308Cfs^a^12	M	
P35(F32)	F/41/3	R/UL	P	B/UL	S/C	NA	Lipid	c.187 + 1G > A	P/M	
P36(F32)	F/40/5	R/UL	D	B/LL	S	NA	Lipid/RVs	c.187 + 1G > A	P/M	
P37(F33)	F/26/9	R/UL	P	R/UL	S	NA	Lipid	c.757 + 1G>T	NA/NA	
P38(F34)	F/44/7	B/UL,LL	P	B/UL	S/C	NA	Lipid	c.757 + 1G > T	NA/NA	
p39(F35)	F/30/3	R/UL	P	R/UL	S/C	+	Lipid	c.757 + 1G>T	P/M	
p40(F36)	F/30/2	R/LL	P	R/UL	S	+	Lipid/RVs	c.750_757del, *p.*R251Pfs^a^53	NA/NA	
P41(F37)	M/17/7	B/LL	P	R/UL	S	+	NA	c.757 + 1G > T	P/M	
P42(F37)	M/21/1	EI	D	B/LL	S	+	NA	c.757 + 1G > T	P/M	
P43(F38)	F/40/10	R/UL	D	R/UL	S/C	NA	Lipid/RVs	c.757 + 1G > T	NA/NA	
P44(F39)	F/27/6	R/UL	P	R/UL	S	NA	Lipid	c.757 + 1G > T	NA/NA	
P45(F40)	F/30/8	R/UL	P	R/UL	S/C	+	Lipid/RVs	c.757 + 1G > T	NA/NA	

Abbreviations: *S/A/D* Sex/onset Age (years)/Duration (years); *JA* Jordan’s anomaly; *R* right; *B* Both; *UL* upper limb weakness; *LL* lower limb weakness, *EI* Exercise intolerance; *P* proximal; *D* distal; *NA* not applicable; *S* skeletal myopathy; *C* cardiomyopathy; *S/C* skeletal myopathy with cardiomyopathy; *AH* asymptomatic hyperCKemia; *RVs* rimmed vacuoles; *P* Paternal; M, Maternal; *PA* patatin domain

^a^Severe limbs refer to the most severely involved limbs in physical examination.

### Muscle MRI features

**Fig. 2:**
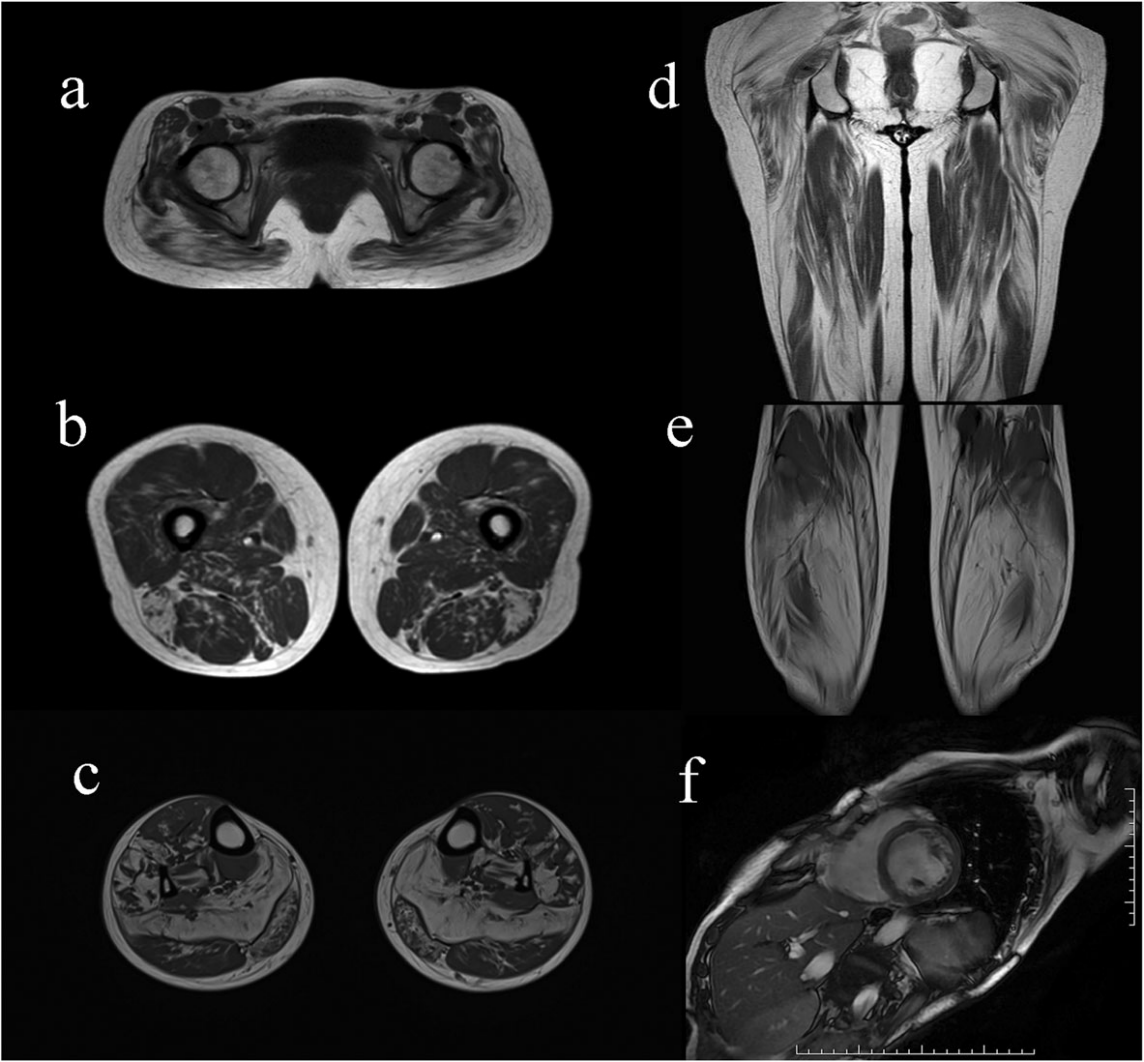
Lower leg muscle and cardiac MRI characteristics in NLSDM patients. **a** MRI revealed diffuse fatty infiltration, predominantly involving the gluteus maximus at the pelvic level. **b**, **d** The long head of the biceps femoris, semimembranosus and adductor magnus were moderately-to-severely affected. The rectus femoris, gracilis and sartorius were relatively preserved. **c**, **e** The soleus and medial head of the gastrocnemius were severely affected. The anterior and posterior tibialis were relatively preserved. (**f**) Cardiac MRI showed dilated cardiomyopathy

**Fig. 3:**
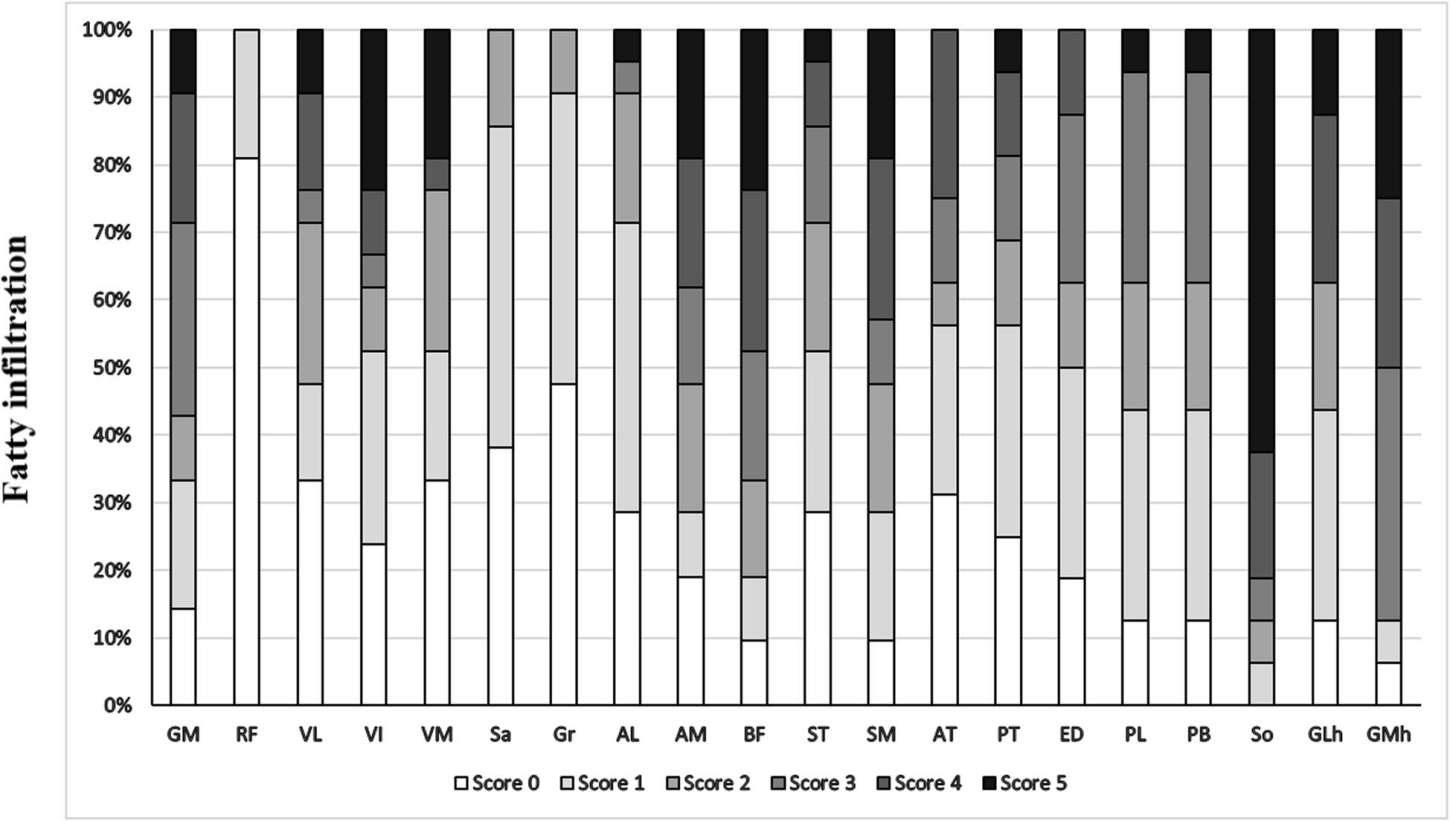
Muscle fatty infiltration score on MRI of upper and lower legs. Abbreviations: GM, gluteus maximus; RF, rectus femoris; VL, vastus lateralis; VI, vastus intermedius; VM, vastus medialis; Sa, sartorius; Gr, gracilis; AL, adductor longus; AM, adductor magnus; BF, biceps femoris long head; ST, semitendinosus; SM, semimembranosus; AT, anterior tibialis; PT, posterior tibialis; ED, extensor digitorum longus; PL, peroneus longus; PB, peroneus brevis; So, soleus, GLh, gastrocnemius lateral head; GMh, gastrocnemius medial head

Twenty-one patients had MRI on the thighs and 16 on the calves. Asymmetric fatty infiltration was observed in five patients, which was prominent on the right limbs of two patients and on the left limbs of three patients. Fatty infiltration appeared in the thighs of 19/21 (90.5%) patients. On MRI scoring analysis, the long head of the biceps femoris, semimembranosus and adductor magnus were the top three severely affected muscles. The rectus femoris, gracilis and sartorius were the top three least involved muscles. Fatty infiltration appeared in the lower legs of 16/16 (100%) patients. The top two severely infiltrated muscles were the soleus and medial head of the gastrocnemius. The top two least infiltrated muscles were the anterior tibialis and the posterior tibialis (Fig. 2, Fig. 3, Additional file 1).

**Fig. 4:**
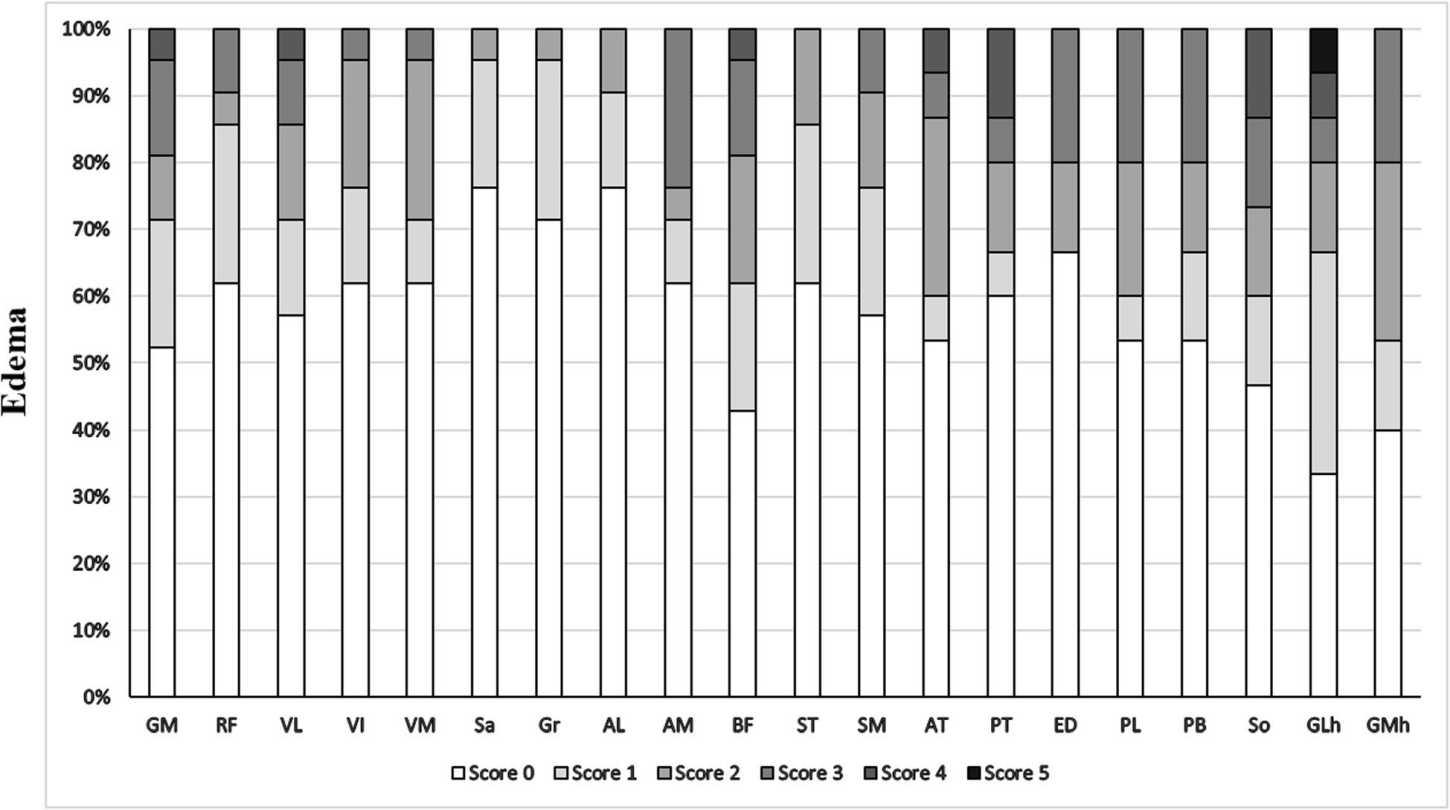
Score of muscle fatty edema on MRI of upper and lower legs. Abbreviations: GM, gluteus maximus; RF, rectus femoris; VL, vastus lateralis; VI, vastus intermedius; VM, vastus medialis; Sa, sartorius; Gr, gracilis; AL, adductor longus; AM, adductor magnus; BF, biceps femoris long head; ST, semitendinosus; SM, semimembranosus; AT, anterior tibialis; PT, posterior tibialis; ED, extensor digitorum longus; PL, peroneus longus; PB, peroneus brevis; So, soleus, GLh, gastrocnemius lateral head; GMh, gastrocnemius medial head

Muscle edema appeared in the thighs of 19/21 (90.5%) patients, and predominantly involved the long head of the biceps femoris, gluteus maximus and the adductor magnus. The sartorius, gracilis and adductor longus were relatively spared. Muscle edema appeared in the lower legs of 16/16 (100%) patients, and predominantly involved the gastrocnemius medial head and the soleus. The anterior tibialis, extensor digitorum longus and peroneus brevis were the three least affected muscles. No asymmetric muscle edema was observed (Fig. 4, Additional file 2).

### Data from muscle pathology and peripheral blood smear

**Fig. 5:**
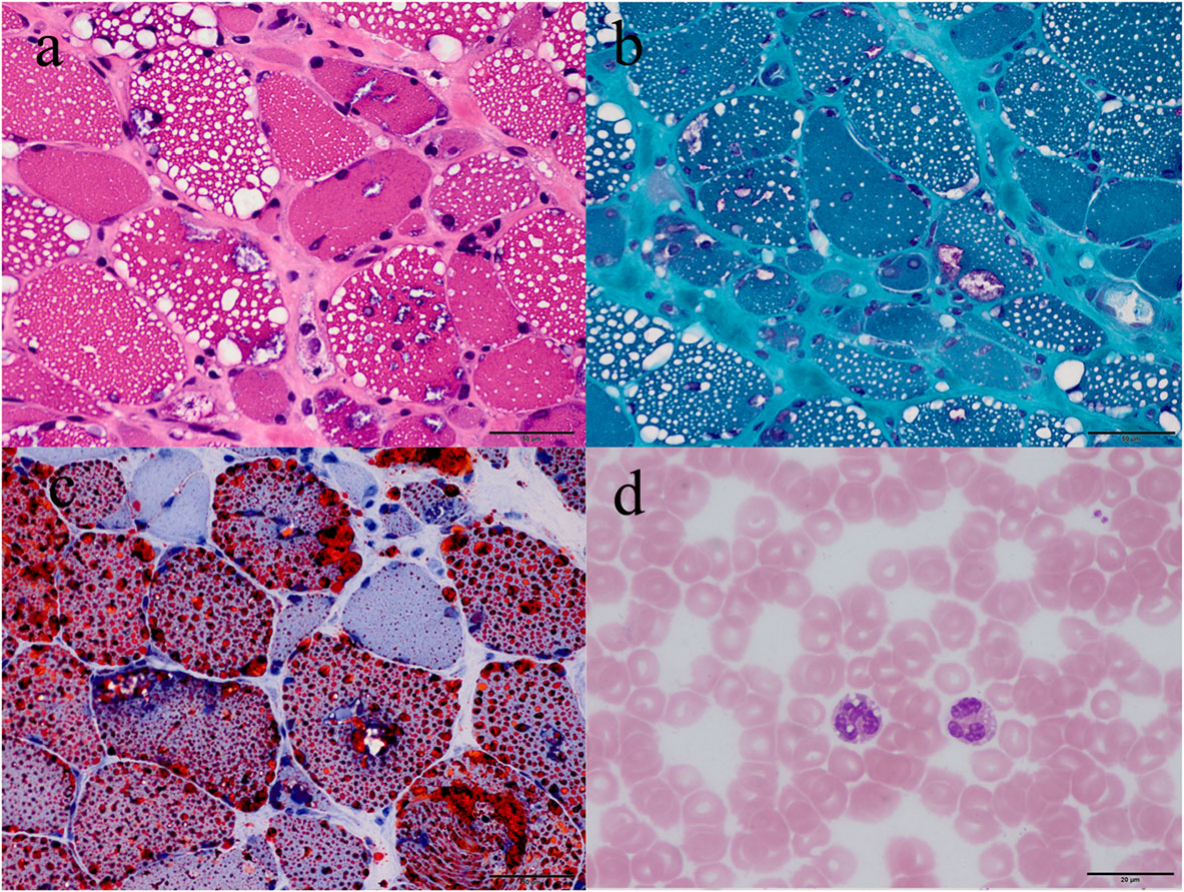
Muscle pathology and peripheral blood smear of NLSDM. **a** Muscle biopsy showing massive vacuoles of various size within myofibers, including some rimmed vacuoles under hematoxylin & eosin staining. **b** Oil red O staining revealed accumulations of a large number of lipid droplets within myofibers. **c** Modified Gomori trichrome staining confirmed rimmed vacuoles. **d** Peripheral blood smear revealed Jordan’s anomaly under Wright’s stain. Scale bar = 50 μm (**a-c**); Scale bar =20 μm (**d**).

Muscle specimens were obtained from 42 patients. The site for biopsy was the biceps in 40 patients, the quadriceps (p45) in one and the deltoid (p39) in one patient. Fibro-adipose replacement was reported in 18 (42.9%) patients, fiber size variation in 24 (57.1%), myofiber necrosis and regeneration in 19 (45.2%) and lipid droplet accumulation in all patients. RVs were identified in 21 (50.0%) patients including 20 specimens from biceps and one from quadriceps. Sixteen patients manifested as proximal myopathy and five patients as distal myopathy. Peripheral blood smear revealed Jordan’s anomaly in all 31 patients tested (Fig. 5, Table 1).

### PNPLA2 gene mutations: frequencies and types

Among 45 patients from 40 families of NLSDM, 33 families were carrying homozygous mutations, while seven families were carrying compound heterozygous mutations. The variants from 14 families have been verified for co-segregation (Table 1). A total of 23 mutations were identified including 11 (47.8%) point mutations, eight (34.8%) deletions and four (17.4%) insertions, which functionally caused 11 (47.8%) frameshift deletions, one (4.3%) in-frame deletion, four (17.4%) splicing mutations, six (26.1%) missense mutations and one synonymous mutation (4.3%). Among the 80 allelic variants from 40 families, *c.*757 + 1G > T (24/80, 30.0%), *c.*245G > A (9/80, 11.3%) and *c.*187 + 1G > A (8/80, 10.0%) were the top three mutations, adding up to over one half of the allelic frequencies. The homozygous *c.*757 + 1G > T was the hotspot mutations. Thirteen patients carrying homozygous or heterozygous mutation of *c.*757 + 1G > T distributed across south and north China (Fig. 1).

Five out of six missense mutations were located on the patatin domain (amino acids 10–179), and the rest one missense mutation and one synonymous mutation were located between the patatin and hydrophobic lipid binding domain (amino acids 309–391). *c.*245G > A, *c.*353 T > C, *c.*356C > A, *c.*434G > A and *c.*516C > G were predicted to be disease-causing in Mutation taster, probably damaging in Polyphen2 and deleterious in SIFT. *c*.749A > C was predicted to be a polymorphism in Mutation taster, benign in Polyphen2 and deleterious in SIFT. Fifteen null mutations (four splicing and eleven deletion/insertion) were judged as pathogenic or likely pathogenic according to ACMG. The homozygous in-frame mutation of *c.*659_667del and the six missense mutations were interpreted as uncertain significance. The synonymous mutation of c.564G > A were also interpreted as uncertain significance since it is a potential alteration of splicing using Human Splicing Finder 3.1 (www.umd.be/HSF3).

**Fig. 6:**
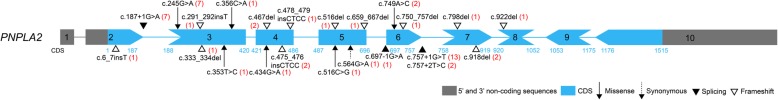
Location of mutations in PNPLA2 gene identified in 45 patients with NLSDM. To accommodate the distribution of mutations, the size of the exons was not represented at scale. To illustrate the reading frame, the exons are schematically represented by boxes with blunt, protrusive or intrusive ends. Nucleotide numbering for all mutations was designated according to the coding DNA reference sequence (CDS) in GenBank Accession number NM_020376 (PNPLA2). Numerals within parentheses indicate the number of patients harboring the mutation

All PNPLA2 defects were listed in Table 1 and shown in Fig. 6.

### Correlation of clinical, pathological, genetic aspects and disease phenotypes

Four phenotypes (skeletal myopathy, cardiomyopathy, skeletal myopathy with cardiomyopathy and asymptomatic hyperCKemia) showed significant differences in disease onset (< 20 years versus ≥20 years old, *p* = 0.003). Asymptomatic hyperCKemia developed before 20 years of age, while cardiomyopathy all developed after 20 years of age. There was also a significant difference in muscle pathology (with RVs versus without RVs, *p* = 0.001) among the four phenotypes. RVs were found in skeletal myopathy with or without cardiomyopathy, but did not appear in pure cardiomyopathy or asymptomatic hyperCKemia. There were no significant differences in gender (male versus female), mutation type (homozygous versus heterozygous mutation) or mutation severity (with one or two null mutations versus missense/in-frame mutations)(Table 2**)**.

**Table 2:** Association of various variables with clinical phenotypes in 45 NLSDM patients

		Phenotypes				*P* value
Variables		S (*n* = 18)	S/C (*n* = 21)	C (*n* = 4)	AH (n = 2)	
Gender	Male	7	6	3	2	0.076
	Female	11	15	1	0	
Age onset	<20y	0	2	0	2	*0.003*
	≥20y	18	19	4	0	
Muscle Pathology	RVs+	13	8	0	0	*0.001*
	RVs-	3	12	4	2	
Mutation type	Homozygous	16	17	4	1	0.374
	Heterozygous	2	4	0	1	
Mutation severity	Missense/in frame	2	4	1	1	0.606
	Null	16	17	3	1	

*S* skeletal myopathy; *C* cardiomyopathy; *S/C* skeletal myopathy with cardiomyopathy; *AH* asymptomatic hyperCKemia; *RVs* rimmed vacuoles

Differences were considered statistically significant at *p* < 0.05

## Discussion

In the present study, the average NLSDM onset age was around 30 years old, with a wide range from 3.5 to 48 years, similar to that previously reported [3–5, 20]. We also found that five patients developed NLSDM before their 20s. Early onset NLSDM has been occasionally reported, with most patients showing a mild phenotype or asymptomatic hyperCKemia [4, 6, 8, 25]. Thus, there is likely a long subclinical period before the initiation of the disease. Unlike skeletal myopathy, cardiomyopathy developed after 20 years of age in all patients, indicating a common but late involvement of the heart. Our study also broadened the clinical spectrum of NLSDM. Skeletal myopathy was the most common phenotype [3, 4, 15, 20, 26]. The right limb was the initial and most severely affected limb in > 60% of patients which justified that the metabolic action disorder in NLSDM, such as impaired fatty acid oxidation [27], is attributed to exercise. Asymmetric right shoulder weakness with intact pharyngeal and respiratory muscles is a useful sign for identifying NLSDM from other muscular disorders [3, 5, 19, 22]. We found predominant distal weakness in approximately 16% of patients. Kaneko [3] and Missaglia [19] also reported high incidence of distal myopathy. From our data, the choice of biopsy sites did not have much influence on muscle pathology such as appearance of lipid droplets or RVs. RVs within myofibers, were found to be associated with the phenotype of skeletal myopathy with or without cardiomyopathy in the present study although it was considered not related to disease onset or mutation sites [28]. RVs might be also an indicator to distal dominant pattern since they are revealed in all of the NLSDM patients with distal myopathy, GNE myopathy and other hereditary distal myopathies. Hepatomegaly was only observed in 5% of our cases, much lower than that previously reported [3, 4, 15].

We found evidence of severe fatty infiltration in the long head of the biceps femoris, long adductor, semimembranosus, gluteus maximus, soleus and medial head of the gastrocnemius in our NLSDM patients, some of which have been previously described in cases [3–5, 8, 12, 15–17, 19, 22]. Since lipid droplets mainly appeared in type 1 fibers, muscle selectivity in NLSDM might be due to the maintenance of standing posture from posterior muscles which contain high proportion of type 1 slow twitch oxidative fibers. We also found that the calf muscles were more severely and diffusely involved compared with the thigh muscles even in proximal myopathy, suggesting lower legs MRI more valuable to NLSDM diagnosis in early stage. Asymmetric fat infiltration of the legs was only observed in six cases, indicating that the muscle asymmetry mainly involved the upper limbs, and tended to become uniform with disease progression. Interestingly, the muscle involvement pattern in our series was similar in both typical and mild phenotype cases, as well as in asymptomatic hyperCKemia [8]. Thus, the muscle MRI pattern was a sensitive indicator for NLSDM diagnosis. Selective posterior muscles involvement is also partly observed in other muscle diseases including desminopathy, fascioscapulohumeral muscular dystrophy, calpainopathy or dysferlinopathy [29, 30], although the relative sparing of the semitendinosus, sartorius and gracilis, and lack of muscle hypertrophy, are helpful for differential diagnosis [31]. Except for inflammatory myopathies, muscle edema is also seen in metabolic myopathy, some types of muscular dystrophy and neurogenic disorders. Unlike fat replacement, muscle edema in our series did not shown a clear and consistent distribution, and may occur secondary to muscle energy failure in NLSDM. However, further studies are required to assess the relationship of muscle edema with disease activity.

Cardiac dysfunction appeared in nearly 40% of our patients, which was comparable to previous studies [3–5, 11, 14]. Cardiomyopathy developed at an advanced stage in the majority of our patients and in other cases [5, 19, 20], or as the sole manifestation. Pure skeletal myopathy and pure cardiomyopathy were also reported in two siblings (p8 and p9, respectively) [10]. Therefore, we suggest cardiomyopathy not only a late stage manifestation but also an independent subtype. Unlike previous reports of artery luminal narrowing [3, 14], one patient (P39) with myocardial infarction on electrocardiogram showed no artery stenosis on coronary angiography. Studies performing cardiac biopsy or autopsy have reported triglyceride droplets within the walls of the coronary arteries in NLSDM [14]. The name of triglyceride deposit cardiomyovasculopathy has thus been proposed by Japanese researcher [14] although is seldom reported [20]. We did not find severe cardiac phenotypes in our series, suggesting an ethnic disparity even between patients of Asian origins. However, male patients showed a trend towards a purer cardiomyopathy than females in this series. In support, Pasanisi [11] reported a higher incidence of cardiac damage in male patients, while estrogens in females were suggested to have a protective effect on the cardiac phenotype [20, 26, 32].

In our series, patient 4 exhibited a missense mutation on the patatin domain and a synonymous mutation. However, we still confirmed the case based on the concurrence of skeletal myopathy and cardiomyopathy, typical MRI pattern and both muscle and blood smear pathology. Janssen [7] also described heterozygous PNPLA2 mutation in the patients showing neutral lipid storage in muscle, Jordan’s anomaly and myopathy. An aberrant mRNA splicing from a synonymous variant, such as in GNE myopathy [33], or inactivation of another allele such as in Duchenne muscular dystrophy, may exist. Our series did not show any association of genotypes with phenotypes. We found frequent homozygous, splicing and frameshift mutations, similar to Japanese patients [9, 13, 14, 18], but different to Italian patients [4]. PNPLA2 mutations were previously reported to be mainly on the lipid binding domain [1, 4, 8, 13, 19, 21]. However, majority of our missense mutations were located on the patatin domain. Cardiomyopathy tends to be found in genetic defects with severe functional damage, whereas missense mutations commonly present sparing of cardiomyopathy because of the partial preservation of lipase activity [19, 34]. Thus, the lack of correlations between genotypes and phenotypes in the present study may be related to the fact that the majority of missense mutations involved the patatin domain, which can also cause dramatic reduction in lipase activity and massive lipid droplets accumulation [19, 34]. Moreover, we confirmed *c.*757 + 1G > T as the hotspot mutation in Chinese patients [22]. *c.*757 + 1G > T, which was previously reported in a small case series of Hmong patients from Southeastern Asian origin [35], were also identified in patients of Han Nationality in our series. Therefore, *c.*757 + 1G > T screening may be useful in suspected NLSDM, although the mutation founder effect requires further confirmation.

## Conclusion

Taken together, we have expanded the clinical and genetic spectrum of NLSDM in a cohort of Chinese patients. The disease onset age and appearance of RVs in our series was associated with the development of various clinical symptoms. Weakness of the right shoulder girdles and the characteristic pattern on muscle MRI were useful for NLSDM diagnosis. Chinese patients tended to present homozygous, splicing, frame shift or missense mutations located on the patatin domain. *c.*757 + 1G > T was the hotspot mutation. However, we did not find a phenotype-genotype relationship, suggesting that the complicated functional damage in NLSDM depends on multiple factors.

## Supplementary information

**Additional file 1.** Muscle fatty infiltration score on leg MRI of 21 NLSDM patients. Score of the fatty infiltration in individual muscles. (L): Left leg was more severe and selected for scoring; (R), Right leg was more severe and selected for scoring. Abbreviations: see Fig. 3.

**Additional file 2.** Muscle edema score on leg MRI of 21 NLSDM patients. Score of the muscle edema in individual muscles. Abbreviations: See Fig. 3.

## Data Availability

The patients data used in this study are included in this publish article and its supplementary files.
